# Molecular Basis of Unequal Alternative Splicing of Human SCD5 and Its Alteration by Natural Genetic Variations

**DOI:** 10.3390/ijms24076517

**Published:** 2023-03-30

**Authors:** Gabriella Orosz, Luca Szabó, Szanna Bereti, Veronika Zámbó, Miklós Csala, Éva Kereszturi

**Affiliations:** Department of Molecular Biology, Semmelweis University, H-1085 Budapest, Hungary; orosz.gabriella@med.semmelweis-univ.hu (G.O.); saboluca@gmail.com (L.S.); szanna.bereti@gmail.com (S.B.); zambo.veronika@med.semmelweis-univ.hu (V.Z.)

**Keywords:** stearoyl-CoA desaturase-5, genetic variation, SNV, alternative splicing, transcript variant, cancer, minigene assay, donor site, acceptor site

## Abstract

Alternative splicing (AS) is a major means of post-transcriptional control of gene expression, and provides a dynamic versatility of protein isoforms. Cancer-related AS disorders have diagnostic, prognostic and therapeutic values. Changes in the expression and AS of human stearoyl-CoA desaturase-5 (SCD5) are promising specific tumor markers, although the transcript variants (TVs) of the gene have not yet been confirmed. Our in silico, in vitro and in vivo study focuses on the distribution of SCD5 TVs (A and B) in human tissues, the functionality of the relevant splice sites, and their modulation by certain single-nucleotide variations (SNVs). An order of magnitude higher *SCD5A* expression was found compared with *SCD5B*. This unequal splicing is attributed to a weaker recognition of the *SCD5B*-specific splicing acceptor site, based on predictions confirmed by an optimized minigene assay. The pronounced dominance of SCD5A was largely modified (rs1430176385_A, rs1011850309_A) or even inverted (rs1011850309_C) by natural SNVs at the TV-specific splice sites. Our results provide long missing data on the proportion of SCD5 TVs in human tissues and reveal mutation-driven changes in SCD5 AS, potentially affecting tumor-associated reprogramming of lipid metabolism, thus having prognostic significance, which may be utilized for novel and personalized therapeutic approaches.

## 1. Introduction

The balance of lipid metabolism in the human body depends on an adequate supply of various saturated (SFA), monounsaturated (MUFA) and polyunsaturated (PUFA) fatty acids (FAs) of different carbon chain lengths, adjusted to the dynamically changing requirements. The MUFA-producing stearoyl-CoA desaturases (SCDs) are endoplasmic reticulum membrane-bound enzymes, which introduce the first double bond at cis-delta9 position into saturated fatty acyl-CoA molecules, mainly palmitoyl-CoA and stearoyl-CoA, to produce palmitoleyl-CoA and oleyl-CoA, respectively [1]. Two isoforms of SCDs have been identified in humans [2]: SCD1, which is indisputably a central regulator in lipid metabolism [3] and SCD5, which raises several, yet unanswered questions.

The two human isoforms show markedly different gene expression patterns. While *SCD1* is mainly expressed in the major organs of lipid metabolism [4], *SCD5* is highly represented in the embryonic and mature brain, pancreas and gonads instead [2,5,6,7]. In addition, increasing evidence suggests that *SCD5* gene expression may be less dependent on lipid factors compared to other mammalian desaturases. In contrast to *SCD1*, the promoter activity of *SCD5* has been shown to be insensitive to several FAs in vitro [6]. Despite the in silico identification of transcription factor (TF) binding sites constituting the PUFA response region (SREBP1, SP1, PGC1*α*, NF1, NFY) in the 5′ regulatory sequence of *SCD5* [8,9], neither diets containing various unsaturated FAs [10], changes in serum lipid levels [11], nor the presence of retinoic acid [12] affected *SCD5* mRNA levels. However, certain promoter polymorphisms of *SCD5* have been proven to significantly reduce the promoter activity in vitro, modify TF binding sites in silico and were associated to metabolic disorders [6].

In recent years, the results of high-throughput whole genome, transcriptome and microRNA sequencing data have directed attention to tumor-related changes in the expression level of *SCD5* [13,14,15,16,17,18,19]; however, the underlying molecular mechanism of these alterations remains to be elucidated. Currently, SCD5 is primarily considered a promising type-specific tumor marker. Nevertheless—based on its catalytic function—its role in metabolic diseases also arises. SCD5 is involved in the development and progression of different cancer types, and the induction of its gene expression by various cellular factors can contribute to tumor cell survival [20]. SCD5-driven reprogramming of FA metabolism has been shown to block epithelial–mesenchymal transition, thereby reducing the probability of metastatic events and tumor malignancy in breast cancer [21] and melanoma [22,23]. Furthermore, SCD5 has also been demonstrated to be a promising predictive biomarker for response to neoadjuvant chemotherapy in breast cancer [16], and a reliable prognostic factor for metastatic uveal melanoma [18].

Alternative splicing (AS) is a fundamental modulator of human gene expression, and it plays a significant role in expanding the diversity of functional proteins. There is increasing evidence that AS has a profound effect on cancer development. The tumor type-specific changes of the splicing process in cancer cells have significant prognostic value, can contribute to the prediction of cancer progression rate, and may be of therapeutic relevance [24]. Along with six other genes, the *SCD5* splicing event was identified as strong prognostic biomarker in a study analyzing the AS profile of kidney renal clear-cell carcinoma [19], however, the exact nature of this splicing process was not defined. These splicing alterations are often associated with the occurrence of natural or cancer driver mutations in genes that encode either core sequence elements or regulators of the splicing machinery, thereby affecting the fine balance of isoforms [24,25,26].

Minigene assay is a reliable in vitro tool for investigating the effect of human variations in AS [27]. After cloning the exon of interest together with the harboring intronic sequences into an appropriate expression vector, the process of AS can be followed either directly by RT-PCR and qPCR, or indirectly through a reporter enzyme [28,29]. The method is excellent for comparing alleles of a splicing site mutation. However, in its original form, it is not suitable for analyzing the biased alternative terminal exon usage of transcript variants (TVs) of one single gene. Certain methodological refinement is required for investigating possible splicing preferences between several alternative donor and/or acceptor sites of the same gene, or for assessing the effect of mutations on relative proportion of the TVs.

Although there are two TVs (A and B) of SCD5 in the NCBI, Ensemble and UniProt databases, they are not distinguished, examined, or even mentioned in scientific publications, with only one exception to our knowledge [30]. Therefore, in the present study, we verified the existence of both *SCD5A* and *SCD5B* forms and assessed their relative mRNA expression and distribution in different human cell lines and tissues. We proved the biased, *SCD5A* dominant acceptor site recognition of the two TVs in silico by prediction, and in vitro by generating and expressing a modified SCD5 minigene construct. Human variations affecting *SCD5A*- and *SCD5B*-specific donor and acceptor splice sites were also tested by the newly developed SCD5 minigene system.

## 2. Results

### 2.1. Confirmation of Transcript Variants A and B of the SCD5 Gene

**Figure 1:**
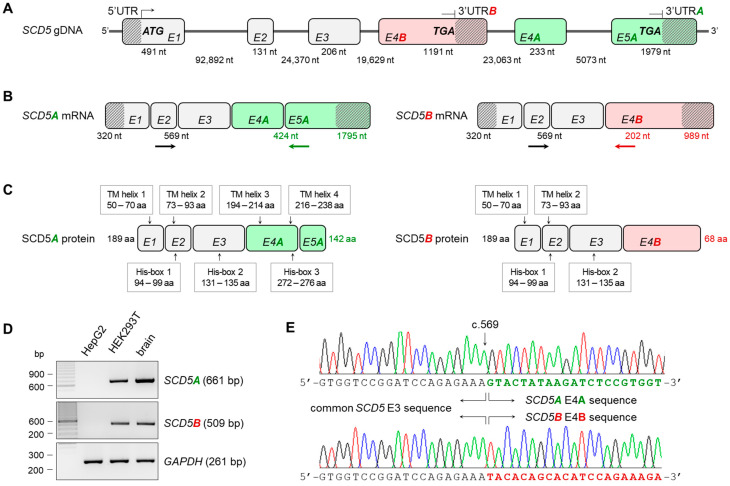
Transcript variants of human *SCD5* gene. Schematic gene (**A**), mRNA (**B**) and protein (**C**) structure of human SCD5A and B transcript variants. Exons are numbered and marked with rectangles. Introns are depicted with straight lines and UTRs with diagonally lined rectangles. The start and stop codons, and the length of introns and exons are also indicated. *SCD5A*- and *SCD5B*-specific and shared exons are shown in green, red and gray, respectively. The position of the putative TM domains and His-boxes in the protein are also marked. Verification of *SCD5A*- and *SCD5B*-specific splicing events by RT-PCR (**D**) and direct sequencing (**E**). RT-PCR primers used to verify A- and B-specific splicing events are indicated by arrows, *SCD5A*- and *SCD5B*-specific and shared primers are shown in green, red and black, respectively. The agarose gel images represent typical results of three independent experiments. The electropherograms show the AS hotspot of the two transcript variants indicating the starting point of the different 3′ ends. (aa: amino acid; nt: nucleotide; TM helix: transmembrane helix; His-box: histidine containing sequence).

The human *SCD5* gene is located on the longer arm of chromosome 4. Its average-length exons are separated by rather long intron sequences of tens of thousands of base pairs. The first three of its six exons are identical for the two TVs, but *SCD5A* contains two more exons (exon 4A, E4A and exon 5A, E5A) (Figure 1B), whereas *SCD5B* has only one (exon 4B, E4B) (Figure 1B), which is localized in the third intron of the other variant (Figure 1A). Accordingly, the two TVs have alternative terminator (AT) sites [31]. Thus, the alternatively spliced forms share the same 5′ region and protein N-terminus, but they differ completely in the 3′ untranslated region (UTR) and in the C-terminal protein structure (Figure 1B,C).

Since variant B is largely neglected in the literature, with one exception [30], we considered it essential to verify that both variants are indeed expressed in vivo. mRNA samples from two human-derived cell lines (HepG2 hepatocellular carcinoma and HEK293T human embryonic kidney) and from human brain tissue were reverse-transcribed into cDNA, and the presence of *SCD5A* and *B* TVs was tested by RT-PCR using primers specific for the common exon 2 and E5A and E4B, respectively, (Figure 1D) and a *GAPDH*-specific reaction as a control. Neither the *SCD5A* nor the *SCD5B* TV could be detected in HepG2 cells; however, both the *SCD5A*- and *SCD5B*-specific fragments were successfully amplified in the HEK293T cell line and in the human brain sample (Figure 1D), proving the existence of AS of the pre-mRNA. This in vivo AS between both E3/E4A and E3/E5A exons was also demonstrated by direct sequencing of the PCR products (Figure 1E).

### 2.2. Expression and Distribution of SCD1, SCD5A and SCD5B mRNAs in Human Tissues

A qPCR-based method was developed and optimized for the independent analysis of *SCD5A* and *SCD5B* gene expression (Figure S1A). The amplification of both *SCD5A* and *SCD5B* was effective, i.e., the amount of the PCR products was exactly doubled in each cycle of the exponential phase of the reaction (slope of −log relC: −3.3436 for *SCD5A* and −3.3649 for *SCD5B*), thus the method proved to be suitable for quantitative comparisons (Figure S1B, Table S1).

**Figure 2:**
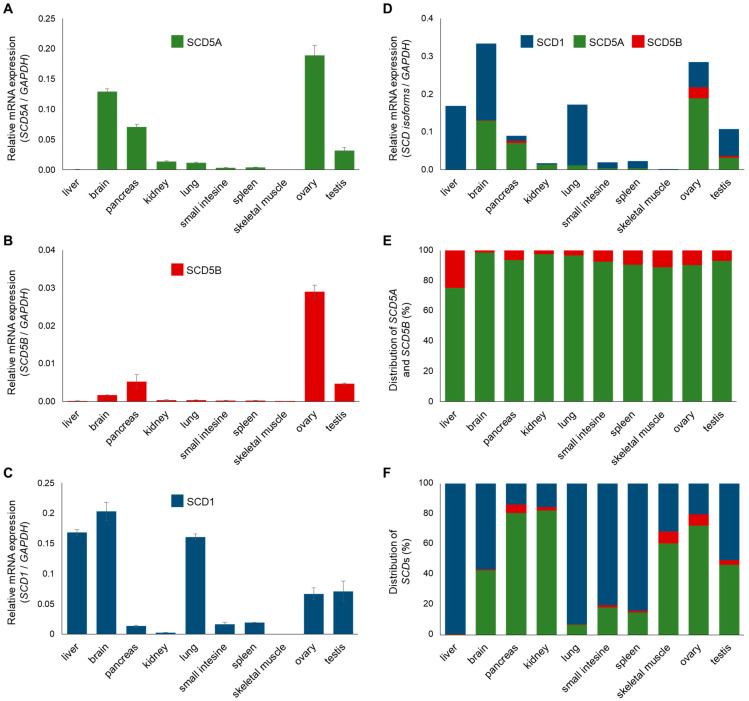
Relative gene expression and distribution of human *SCD*s in different tissues. Relative mRNA expression of *SCD5A* (**A**), *SCD5B* (**B**), *SCD1* (**C**) and cumulated relative *SCD* gene expression (**D**) were measured in ten different human tissues by qPCR, as described in Section 4. *GAPDH* gene expression served as a control. The distribution of *SCD5* transcript variants (**E**) and all three *SCD* mRNAs (**F**) in human tissues is represented on a percentage scale, where total *SCD5* or total *SCD* expression is considered as 100 percent. Experiments were performed in triplicate. In charts (**A**–**C**), data are shown as the mean values ± S.D. The S.D. values for sections (**D**), (**E**) and (**F**) are presented in Tables S2, S3 and S4, respectively.

The tissue distribution of *SCD1* is well characterized [4]. Similar data are also available for *SCD5*, but without distinguishing its two TVs [2,5,6,7]. We, therefore, examined the mRNA expression of *SCD1*, *SCD5A* and *SCD5B* in ten human tissues (liver, brain, pancreas, kidney, lung, small intestine, spleen, skeletal muscle, ovary and testis; Figure 2). As expected, *SCD5A* showed higher expression in the brain, pancreas and gonads (Figure 2A), while *SCD1* was markedly expressed in the liver and lung, in addition to the brain and gonads (Figure 2C). The expression profile of *SCD5B* was nearly identical to that of *SCD5A*, except for a much lower expression of *SCD5B* in the brain compared to variant *A* (Figure 2B).

Comparing the total *SCD* mRNA expression of ten human tissues, the brain and ovary proved to contain the largest amounts of delta9 desaturases (Figure 2D). Significant *SCD* expression was also measured in the liver, pancreas, lung and testis. The expression level of delta9 desaturases was found to be low, but measurable in the kidney, small intestine and spleen, but negligible in skeletal muscle. Since commonly used internal control genes, such as *GAPDH*, may be expressed to a different extent in different tissues, the relative *SCD* mRNA expression was also determined by using two other controls, i.e., *Tubulin* (Figure S2A) and *Actin* (Figure S2B, Table S5), which resulted in very similar *SCD* mRNA expression patterns. It is noteworthy that the total *SCD* isoform composition was completely different in the tissues investigated (Figure 2F). While *SCD1* is by far the major isoform in the liver, lung, small intestine and spleen, the pancreas, kidney and ovary are characterized by a marked *SCD5* predominance, and the presence of the two isoforms is balanced in the brain, skeletal muscle and testis (Figure 2F).

When analyzing the tissue distribution of the two TVs of *SCD5*, it was revealed that *SCD5B* is significantly less expressed, and its level is at least one order of magnitude lower than that of *SCD5A* in all the tissues examined (Figure 2E).

### 2.3. In Silico Analysis of Splice Sites in the Alternative Terminator Region of SCD5

**Figure 3:**
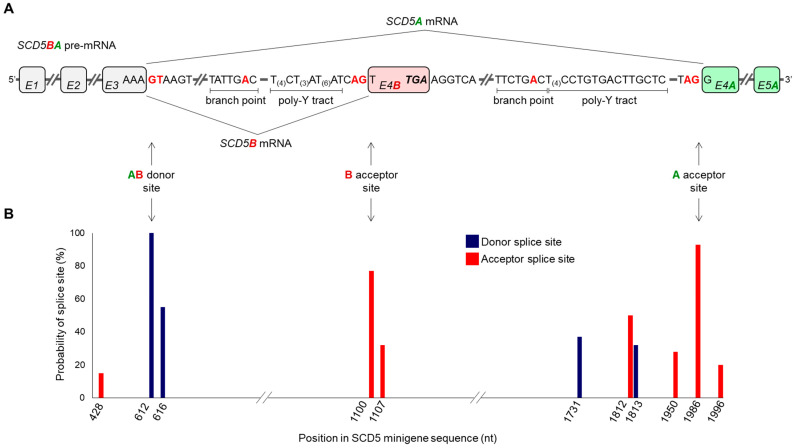
*SCD5A*- and *SCD5B*-specific splice site analysis in silico. (**A**) Schematic representation of *SCD5* pre-mRNA. The splice sites, branch points and poly-Y tracts harboring sequences required for the formation of the alternative terminator of *SCD5* are presented. *SCD5A*- and *SCD5B*-specific and shared exons are shown in green, red and gray, respectively. Core nucleotides of the splice sites are marked in red. Possible AS events are indicated by arrows. (**B**) Probabilities of AB donor site and A and B acceptor sites were predicted by NetGene2-2.42, as indicated in Section 4 and plotted over the position in SCD5 minigene sequence. (nt: nucleotide; poly-Y tract: poly-pyrimidine tract).

Since the two SCD5 TVs are produced from the same gene with extensively identical 5′ segments (i.e., 5′ UTR and three exons) (Figure 1A), their unequal tissue distribution (Figure 2E) can hardly be due to different transcriptional regulation. Analyzing the sequence of the pre-mRNA transcribed from the *SCD5* gene, we identified the donor and acceptor sites that are essential for the formation of the alternative terminator region. Figure 3A depicts the sequences delimiting the common donor site at the border of the third exon and intron (hereafter referred to as AB donor site) and the variant-specific acceptor sites recognized during *SCD5A* or *SCD5B* splicing (hereafter referred to as A acceptor site or B acceptor site).

The possibility arises that the unequal splicing of *SCD5* may be due to differential recognition of A and B acceptor sites by the splicing machinery. To test this hypothesis in silico, we analyzed approximately 500-base-pair-long sequences harboring the AB donor and the A and B acceptor sites by NetGene2-2.42 online prediction program package [32]. It revealed 100% probability for the shared AB donor site in *SCD5* pre-mRNA (Figure 3B), and predicted a 93% and 77% probability for the A and B acceptor sites, respectively, consistent with higher expression of *SCD5A* compared to *SCD5B* in human tissues (Figure 2E). It indicates the method’s reliability that no additional donor or acceptor sites were identified above the 60% threshold set by the program.

### 2.4. Minigene Assay of Splice Sites in the Alternative Terminator Region of SCD5

**Figure 4:**
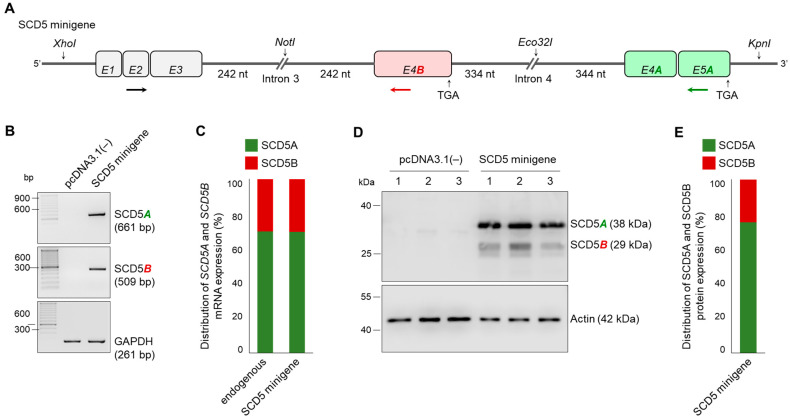
Minigene analysis of unequal *SCD5* alternative splicing in HEK293T cells. (**A**) Schematic structure of SCD5 minigene construct. Exons are numbered and marked with rectangles. The length of the segments cloned from 5′ and 3′ regions of introns 3 and 4 are also shown. The stop codons and restriction endonuclease cleavage sites are indicated. RT-PCR primers used to verify A- and B-specific splicing events are indicated by arrows. *SCD5A*- and *SCD5B*-specific and shared primers are shown in green, red and black, respectively. (**B**) Verification of AS in HEK293T cells transfected with the SCD5 minigene plasmid. The RT-PCR products specific for *SCD5* transcript variants were separated and visualized on 2% agarose gel. *GAPDH* served as an endogenous control. Representative results of four independent experiments are shown. (**C**) The distribution of endogenous and SCD5 minigene-derived *SCD5A* and *SCD5B* expression was measured by qPCR and is represented on a percentage scale. Experiments were performed in triplicate. The S.D. values are shown in Table S6. (**D**) SCD5A and SCD5B protein levels were detected by immunoblotting in samples prepared from pcDNA3.1(−) and SCD5 minigene transfected cells. HEK293T cells were harvested and processed 24 h after transfection. Aliquots of cell lysates (5 µg) were loaded on 15% SDS-polyacrylamide gel, transferred to Immobilon-P membrane, and detected by SCD5-specific antibody. Actin was measured as a loading control. Representative immunoblots of six independent experiments are shown. (**E**) The band intensities of immunoblots were quantified by densitometry and SCD5 protein distribution is represented on a percentage scale. The S.D. values are shown in Table S6.

To investigate whether acceptor sites with different predicted probabilities actually represent different degrees of splicing, we developed a pcDNA3.1(−)-based minigene system, in which the two acceptor sites can be compared simultaneously in the same cell. The schematic structure and exact sequence of the SCD5 minigene are shown in Figure 4A and Figure S3, respectively.

Briefly, the first three exons of the *SCD5* gene, as well as the exons E4A and E5A without introns, were cloned into the expression vector. E4B was cloned together with the adjacent intron sequences. Due to their length, the entire introns 3 and 4 cannot be cloned into a vector, thus, only segments important for splicing were placed in the positions corresponding to the SCD5 gene. In the case of intron 3, a section with a length of 242 nucleotides from the 3′ end and 334 nucleotides from the 5′ end were inserted, while in the case of intron 4, the fragments were 242 and 344 nucleotides long (Figure 4A). Both *SCD5A*- and *SCD5B*-specific splicing events were confirmed by RT-PCR in the RNA samples isolated from HEK293T cells transiently transfected with the SCD5 minigene construct. Neither *SCD5A* nor *SCD5B* could be detected in cells transfected with an empty vector, while both TVs were amplified in samples carrying the SCD5 minigene (Figure 4B). *GAPDH* served as an endogenous and technical control. Quantitative comparison of the two parallel splicing processes was performed using *SCD5A*- and *SCD5B*-specific qPCR, which revealed an unequal distribution of the two TVs, i.e., the minigene-derived *SCD5B* comprised about one third of the total amount of *SCD5*, which fully corresponds to the distribution of endogenous mRNA *SCD5* measured in HEK293T cells (Figure 4C). It should be noted that in some cases, a larger product was also amplified in the *SCD5A*-specific RT-PCR (Figure S4C). Although there is no donor site at the 3′ end of E4B, in the minigene system, introns 3 and 4 can be excised simultaneously by two independent splicing events, so that the E4B exon remains in the *SCD5A* sequence, thereby creating an artificial *SCD5BA* transcript (Figure S4A,B). Although even the very small amount of this BA hybrid byproduct could serve as a template in the qPCR measurements due to the location of the primers (Figure S4B), this would increases the amount of both *SCD5A* and *SCD5B* PCR products by the same extent (Figure S4D), and hence it may only cause a slight underestimation of the difference between the two TVs.

The proper functioning of the SCD5 minigene, and the unequal splicing process with the dominance of SCD5A variant were also confirmed at the protein level by immunoblotting (Figure 4D). The amount of SCD5A protein was approximately three times that of SCD5B (Figure 4E).

### 2.5. Effect of Natural Genetic Variants of SCD5A- and SCD5B-Specific Donor and Acceptor Sites on Alternative Splicing

Human SNVs (single-nucleotide variants) affecting the *SCD5* AB donor site and the A and B acceptor sites were selected from the NCBI and Ensembl databases. We only examined variations that had been detected in at least two populations or two cases in any population.

#### 2.5.1. In Silico Analysis

We examined a total of eight sequence variations of six SNVs, of which the position and main characteristics are summarized in Table 1.

**Figure 5:**
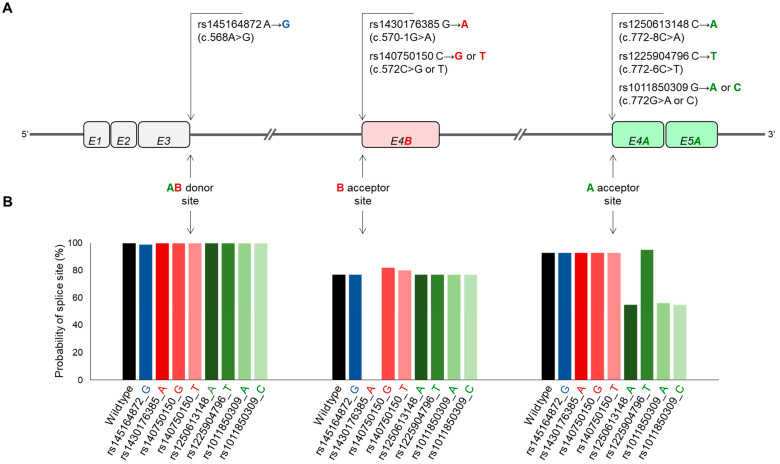
Effect of natural sequence variations predicted by the NetGene2-2.42 program on the recognition probability of *SCD5A*- and *SCD5B*-specific donor and acceptor sites. (**A**) Position of SNVs affecting donor and acceptor sites critical for alternative splicing in the *SCD5* gene. Exons are numbered and marked with rectangles. *SCD5A*- and *SCD5B*-specific, as well as shared exons are shown in green, red and gray, respectively. In addition to localization, nucleotide exchanges caused by SNVs are also indicated. (**B**) Probabilities of donor and acceptor sites for *SCD5* sequence variants predicted by NetGene2-2.42, as indicated in Section 4. Probabilities are plotted as a function of sequence variations at a given donor or acceptor site. The black, blue, green and red columns in the diagram refer to wild type, AB donor site SNV, B acceptor site variants and A acceptor site variants, respectively.

A single missense variation (rs145164872_G, Figure 5A) was identified at the AB donor site that met the selection criteria described in Section 4.

This results in a Lys→Glu or Asn→Asp amino acid exchange, depending on whether SCD5A- or SCD5B-specific splicing event occurs (Table 1). According to the Variant Effect Predictor (VEP, see Section 4) online prediction tool, this SNV can have only a moderate effect on the protein in both cases. At B acceptor site, two SNVs with three sequence variants can influence the AS (Figure 5A). Due to its intronic location, rs1430176385_A does not affect the amino acid sequence, but it appears as a high-impact acceptor variant in the VEP-based prediction. In addition to the wildtype, rs140750150 has two allelic variations (rs140750150_T and rs140750150_G) that result in amino acid changes (Thr→Ile and Thr→Arg, respectively) of moderate predicted effect (Table 1). Four sequence variants of three SNVs in the A acceptor site can modify the AS (Figure 5A). Two of these, rs1250613148_A and rs1225904796_T, affect intronic sequences with low predictive impact (Table 1). The C variant of rs1011850309 is a missense mutation (Lys→Asn) with a moderate effect predicted in silico, while the A allele of the same position is a synonymous variant with low predicted impact on splicing (Table 1).

The extent of the potential effect of the above-mentioned eight sequence variations on the *SCD5* AB donor and A or B acceptor sites was also analyzed in silico by the online available splice site prediction program, NetGene2-2.42 (Figure 5B). The variant rs145164872_G did not significantly affect the probability of the AB donor site, reducing it by only 1%. In contrast, the effect of the rs1430176385_A variant was pronounced. In line with the high impact predicted by VEP (Table 1), according to NetGene2-2.42, this sequence variation not only reduces, but completely eliminates the B acceptor site (Figure 5B). At the same time, both the G and T alleles of rs140750150, albeit slightly, increase the probability of this splice site (to 82% and 80% vs. 77%, Figure 5B). Three of the four sequence variations of the A acceptor site (rs1250613148_A, rs1011850309_A and rs1011850309_C) significantly reduce the probability of the splice site (to 55%, 56% and 55% vs. 93%, respectively), while rs1225904796_T minimally increases it (to 95% vs. 93%, Figure 5B).

#### 2.5.2. In Vitro Analysis

**Figure 6:**
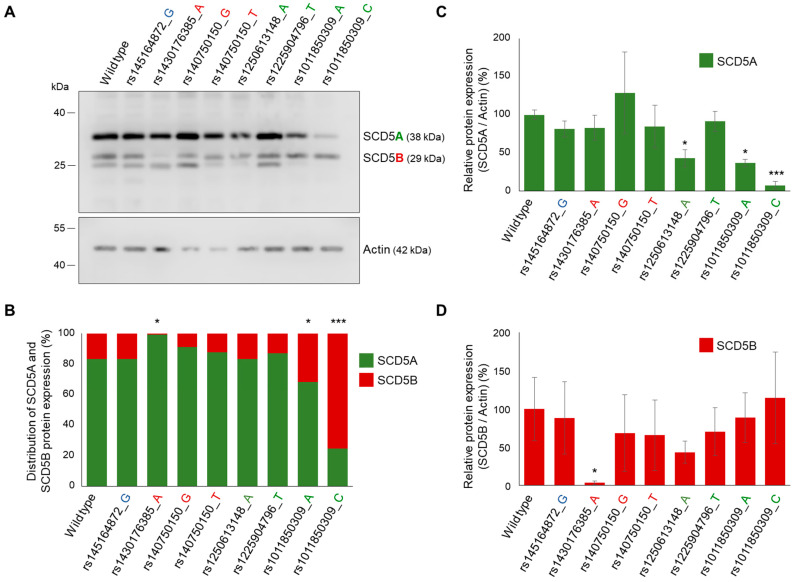
Effect of SNVs on unequal splicing of SCD5 in vitro. HEK293T cells were harvested and processed 24 h after transfection with SCD5 minigene variants. (**A**) Aliquots of cell lysates (5 µg) were loaded on 15% SDS-polyacrylamide gels, transferred to Immobilon-P membrane and SCD5 was detected with an anti-SCD5 antibody. Actin was measured as a loading control. Representative immunoblots of three independent experiments are shown. (**B**) The band intensities were quantitated by densitometry and the distribution of SCD5 transcript variants is represented on a percentage scale. The S.D. values are shown in Table S7. Relative SCD5A/Actin (**C**) and SCD5B/Actin (**D**) ratios are shown as bar graphs. Data are presented as the mean values ± S.D. Statistical analysis was performed with the Tukey–Kramer multiple comparisons test. * *p* < 0.05; *** *p* < 0.001.

In silico predictions of splicing site SNVs were also tested in vitro, at both mRNA and protein levels. The allelic variants were introduced into the SCD5 minigene construct by site-directed mutagenesis, and SCD5A and SCD5B expression was monitored by qPCR (Figure S5) and immunoblotting (Figure 6) in samples isolated from transiently transfected HEK293T cells. According to in silico predictions, the AB donor site variant had no effect on the distribution of the SCD5 forms.

However, the distribution showed marked differences from the wildtype in the case of certain sequence variations affecting the acceptor sites. As expected, the variant rs1430176385_A, which was considered highly effective by VEP and predicted to abolish the B acceptor site by NetGene2-2.42, significantly reduced the proportion of *SCD5B* mRNA expression (*SCD5A*/*SCD5B* for wt: 75%/25%; for rs1430176385_A: 87%/13%, Figure S5). This effect was even more pronounced at the protein level, as the SCD5B protein was practically undetectable in the case of the rs1430176385_A variant (Figure 6A), resulting in a 99%/1% SCD5A/SCD5B ratio (Figure 6B). It is also evident that the shift towards SCD5A observed in the case of rs1430176385_A results solely from a marked reduction in the SCD5B form (Figure 6D), while the level of SCD5A remained unchanged (Figure 6C).

All the three A acceptor site SNVs, which significantly decreased the splice site probability (rs1250613148_A, rs1011850309_A and rs1011850309_C) were seen to reduce the amount of SCD5A protein in the transiently transfected HEK293T cells compared with the wildtype (Figure 6A,C). However, a significant shift in the SCD5A/B distribution, at both mRNA and protein levels, was only observed in the case of rs1011850309_C, where the proportion of *SCD5B* mRNA increased from 25 to 37% (Figure S5), and that of SCD5B protein elevated from 16 to 75% (Figure 6B). It is noteworthy that this shift in the distribution of TVs is primarily due to a significant reduction in SCD5A (Figure 6C), without any obvious change in SCD5B protein (Figure 6D). The effect predicted by NetGene2-2.42 for the A allele of the same SNV (rs1011850309_A) was also detected at the protein level (Figure 6B), although to a lesser extent. The proportion of SCD5B protein increased from 16 to 32%, and it was again attributable to a change in SCD5A protein indicative of hindered *SCD5A* splicing (Figure 6C,D).

## 3. Discussion

Despite the recent progress in research on the transcriptional, nutritional and hormonal aspects of SCD5 regulation [33], the existence of its two transcript variants remained elusive and their actual expression in different tissues awaited proof. The present work provides clear evidence for the transcription of *SCD5B* variant (Figure 1); however, it also reveals that the *SCD5B* mRNA level is significantly, by at least one order of magnitude, lower than *SCD5A* in all human tissues examined (Figure 2). The largely diverse *SCD5A* and *SCD5B* mRNA expressions in vivo may be due to a marked difference between the functionality of the splice sites required to form the alternative terminator regions of these TVs. To test this assumption, we created a special minigene system containing the crucial sections of both introns necessary for AT formation, as well as the original sequences around the exon–intron boundaries, thus allowing for a comparison of the recognition probabilities of the two acceptor sites (Figure 4A). Although our in vitro findings (Figure 4) were in line with the results of our in silico analysis (Figure 3) and confirmed that the B acceptor site is less likely to be recognized during RNA processing, the 3:1 SCD5A/SCD5B ratio, which was detected both at the mRNA and protein levels in the cells expressing the minigene, exceeded the 10–100:1 ratio generally observed in vivo (Figure 2E). The possible contribution of the different 3′ UTRs of the two TVs cannot be ruled out, as this region is well known to play a significant role in mRNA stability [34]. However, no studies have addressed this aspect so far. Furthermore, the formation of the two protein variants may also be influenced by their different C-termini. SCD1, the other human isoform, has a short half-life, in which the role of its N-terminal PEST domain has been well established [35,36,37,38]. Although the absence of the PEST sequence may provide a longer half-life for SCD5 than SCD1, the distinct C-termini of its two TVs may result in different C-degron-dependent degradation pathways, and thus significantly affect the intracellular ratio of SCD5A and SCD5B in vivo [39].

Although the characteristic gene expression of both *SCD1* and *SCD5* has been investigated separately in human tissues [2,4,5,6,7,33], no experimental data are available on the distribution of the two isoforms within a given tissue, nor have the two TVs of *SCD5* been distinguished in the studies. We analyzed the mRNA expression of *SCD1*, *SCD5A* and *SCD5B* in ten human tissues. Since the level of certain endogenous controls may vary between tissue types, we used three different control genes and detected very similar expression patterns, which highly increases the reliability of our results (Figure 2D and Figure S2). Although the hepatic delta9 desaturase expression is undoubtedly high, its extreme value, when normalized to *Tubulin* (Figure S2A), is likely due to the relatively low and variable expression of this control gene in the liver [40,41]. In addition to comparing between tissues, we also focused on determining the proportion of *SCD1*, *SCD5A* and *SCD5B* within each tissue (Figure 2F). It has become clear that the isoform distribution of tissues that express *SCD*s to a high (liver, brain, pancreas, lung and gonads) or low extent (kidney, small intestine, spleen and skeletal muscle) varies widely. Liver, lung, small intestine and spleen mainly express *SCD1*, and the pancreas, kidney and ovary are dominated by *SCD5*, while the two genes show balanced expression in brain, skeletal muscle and testis. Even though the SCDs catalyze the same reaction, their tissue-specific distribution and varying proportion suggest different functions, the nature of which requires further investigation.

Besides the potential impact on expression levels, the different C-termini of the two SCD5 TVs can also lead to versatile alterations in protein behavior. It is well known that the process of alternative splicing, including the formation of alternative terminal exons, is frequently dysregulated in cancers leading to changes in oncogene and tumor suppressor gene expressions [42]. At the same time, protein versions of different C-terminal exons can offer regulatory potentials in healthy cells, since alternative splicing can change sequences that affect intracellular localization, post-translational modification or association with other proteins [43,44], thus allowing the proteins to gain different functions even in different compartments [45]. In addition, AS can greatly affect enzymatic properties [46], substrate binding [47] and protein–protein interactions [48].

The amino acid sequence of human SCD5A indicates four transmembrane domains, similarly to other SCD isoforms. The three histidine clusters considered important to fatty-acyl desaturase activity are also identified in SCD5A [2]. It is notable, that only two of the four transmembrane domains and two of the three histidine boxes are located in the identical N-terminus of the two SCD5 TVs, and although only predicted data are currently available on the structure of the C-terminal sequence of SCD5B (Figure 1C), a partial absence of these structural elements may suggest a different function (https://services.healthtech.dtu.dk/services/TMHMM-2.0/ (accessed on 20 February 2023), [49]).

As it became apparent that coordinated splicing networks are involved in the regulation of tissue and organ development, and also contribute to every hallmark of cancer progression, and hence their assessment has prognostic value, splice site SNVs have advanced to an emerging area of research. The link between alternative splicing site mutations and tumor formation is undoubted [24], and it has also been evidenced that 9–11% of pathogenic mutations underlying rare genetic disorders modify the AS process [50]. AS-dependent isoforms of certain genes can possess very different functions [51,52,53]. Despite the abundance of bioinformatical tools available, predicting the effects of SNVs on splicing still remains a challenging task [54]. In addition, the in silico analysis alone cannot be conclusive, and it is necessary to confirm the predicted effect at least in vitro [55]. Here, we predicted and experimentally tested not only the strength of *SCD5A*- and *SCD5B*-specific donor and acceptor splice sites, but also the effect of their SNVs on variant proportions. Our in silico and in vitro results were in very good agreement in both cases, i.e., the B acceptor site of lower-predicted probability indeed resulted in a lower level of *SCD5B* mRNA (Figure 3 and Figure 4) and the rs1430176385 SNV, which was predicted as deleterious by both NetGene and VEP, and completely prevented SCD5B-specific splicing in our cellular model. The in silico and in vitro data were also consistent for the SCD5A-reducing effect of rs1250613148 and rs1011850309 SNVs, both indicating a decreased recognition of the affected A acceptor site system (Table 1, Figure 5 and Figure 6, Figure S5).

The importance of alternative splicing disturbances in human diseases, such as liver disease [56], cardiovascular disease [57] or cancer [58], is evident, regardless of whether the specific AS shift is a cause or a consequence of the primary condition. The SCD5 splicing event that is in the focus of our study has been identified previously as a significant prognostic marker [16,22] of malignancy [22], treatment response [16] and metastasis in cancer [18,21,23]. We felt, therefore, imperative to fill the gaps in the knowledge of the two TVs of SCD5 and on the determinants of the AS of these gene products, and undertake in silico and in vitro functional analysis of splicing alterations linked to natural genetic variations. The collected data on the SCD isoform expression pattern in various human tissues, and an insight into the impact of SNVs affecting the strength of crucial splice sites in *SCD5* pre-mRNA, and thus on the outcome of its AS, are valuable because they can improve prognosis and help in the development of personalized treatment.

## 4. Materials and Methods

### 4.1. Chemicals and Materials

Culture media and supplements were purchased from Thermo Fisher Scientific (Waltham, MA, USA). Bovine serum albumin, HepG2 and HEK293T cells were purchased from Sigma-Aldrich (St. Louis, MO, USA). Polyclonal primary antibody against SCD5 was obtained from Invitrogen (Carlsbad, CA, USA). Actin-specific polyclonal antibody and secondary antibodies were obtained from Cell Signaling (Danvers, MA, USA). Human tissue RNAs were purchased from Thermo Fisher Scientific (Waltham, MA, USA) and Zyagen Laboratories (San Diego, CA, USA). All chemicals used in this study were of analytical grade. All experiments and measurements were carried out by using Millipore ultrapure water.

### 4.2. Web-Based Tools for In Silico Analysis

The studied SNVs were selected from the NCBI and the Ensembl databases. Mutations affecting the donor and acceptor splicing sequences of the third and fourth introns of the *SCD5* gene were included in the present study if they were identified in at least two different populations. The in silico impact of the selected sequence variants was predicted using the Variant Effect Predictor (https://www.ensembl.org/Homo_sapiens/Tools/VEP/, accessed on 12 Januray 2023, [59]) and NetGene2-2.42 (https://services.healthtech.dtu.dk/service.php?NetGene2-2.42, accessed on 18 July 2022, [32]) online prediction programs.

### 4.3. Expression Plasmid Construction and Mutagenesis

The coding regions of *SCD5A* and *SCD5B* were amplified from human ovary cDNA by iProof™ High-Fidelity DNA Polymerase (Bio-Rad, Hercules, CA, USA), according to the manufacturer’s protocol. The purified DNA fragments were cloned into the pcDNA3.1(−) expression vector between the *Xho*I and *Kpn*I restriction sites. The SCD5 minigene expression plasmid was generated in the pcDNA3.1(−) vector. The construct was designed to include the possible donor and acceptor sites of the third and fourth introns relevant for the alternative splicing of *SCD5A* and *SCD5B* TVs, the branch points, and their flanking sequences. A detailed description of the cloning procedure is presented in Figure S6. Briefly, first, the DNA section required for the entire minigene was amplified in five parts using recombinant *SCD5A* plasmid or genomic DNA as a template. Then, the first two and the last two overlapping PCR products were combined by overlapped extension PCR. The three DNA fragments containing the three common and the three different exons, as well as the 5′ end, 3′ end and branch point regions, were successively cloned into the pcDNA3.1(−) expression plasmid using *Xho*I, *Not*I, *Eco*32I and *Kpn*I restriction sites. The sequence of the cloning primers is shown in Table S8. The studied human natural variants were generated by Q5^®^ Site-Directed Mutagenesis Kit (New England BioLabs, Ipswich, MA, USA). The mutagenic primers are listed in Table S9. All constructs were verified by Sanger-sequencing.

### 4.4. Cell Culture and Transfection

Human embryonic kidney (HEK293T) and hepatocellular carcinoma (HepG2) cells were cultured in 12-well plates (1 × 10^6^ cells per well) in Dulbecco’s modified eagle medium (DMEM), supplemented with 10% fetal bovine serum and 1% penicillin/streptomycin solution, at 37 °C in a humidified atmosphere containing 5% CO_2_. Cells were transfected with 1 μg SCD5 minigene plasmids using 3 μL Lipofectamine 3000, supplemented with 2 µL P3000 (Invitrogen, Carlsbad, CA, USA) in 1 mL DMEM, respectively. Cells were harvested and processed 24 h after transfection.

### 4.5. Preparation of Cell Lysates

Cell lysates were prepared for immunoblot analysis by removing the medium and washing the cells twice with PBS. The RIPA lysis buffer (100 µL) (0.1% SDS, 5 mM EDTA, 150 mM NaCl, 50 mM Tris, 1% Tween 20, 1 mM Na_3_VO_4_, 1 mM PMSF, 10 mM benzamidine, 20 mM NaF, 1 mM pNPP and protease inhibitor cocktail) was added to each well, and the cells were scraped and briefly vortexed. After 45 min incubation on ice, the lysates were centrifuged for 15 min at maximum speed in a benchtop centrifuge at 4 °C, to remove cell debris. The protein concentration of the supernatant was measured with Pierce^®^ BCA Protein Assay Kit (Thermo Fisher Scientific, Waltham, MA, USA), and the samples were stored at −20 °C until downstream analysis.

### 4.6. Immunoblot Analysis

Aliquots of cell lysates (5 μg protein per lane) were analyzed by SDS-PAGE on 15% Tris-glycine minigels, and transferred onto Immobilon-P membranes (Millipore, Billerica, MA, USA). Primary and secondary antibodies were applied overnight at 4 °C and for 1 h at room temperature, respectively. Horseradish peroxidase (HRP)-conjugated goat polyclonal anti-actin (Cell Signaling, Danvers, MA, USA, sc-1616) antibodies were used at 1:1000 dilution. SCD5 was detected with a rabbit polyclonal antibody (Invitrogen, Carlsbad, CA, USA, PA5-89006), used at a dilution of 1:2000, followed by HRP-conjugated goat polyclonal anti-rabbit IgG (Cell Signaling, Danvers, MA, USA, 7074S) at a dilution of 1:2000. HRP was detected by C-DiGit^®^ Blot Scanner (LI-COR, Lincoln, NE, USA) using the SuperSignal West Pico Chemiluminescent Substrate (Thermo Fisher Scientific, Waltham, MA, USA).

### 4.7. RNA Isolation, cDNA Synthesis and RT-PCR

Total RNA was purified from transfected HEK293T and HepG2 cells by using RNeasy Plus Mini Kit (Qiagen, Germantown, MD, USA) following the manufacturer’s instruction. Possible DNA contamination was removed by DNase I treatment using RNAqueous^®^-4PCR Kit (Invitrogen, Carlsbad, CA, USA). cDNA samples were produced by reverse transcription of 0.5 µg DNA-free RNA, using the SensiFAST^TM^ cDNA Synthesis Kit (Meridian Bioscience, Memphis, TN, USA). Reverse-transcription PCR was performed in 10 µL final volume containing 1 µL cDNA template, 0.2 mM dNTPs, 1× PCR Buffer, 1× Q-Solution, 0.025 U/µL HotStarTaq DNA Polymerase (Qiagen, Hilden, Germany), 1 µM forward and reverse primers. *SCD5A* and *SCD5B* sequences were amplified by a commonsense primer (5′-CGC TCT GGG TGT GAC A-3′), together with *SCD5A* (5′-CCC CAG CCA GCA CAT GAA AT-3′)- or *SCD5B* (5′-CCT CCA GGG ACA CAG AAA GAG-3′)-specific antisense primer. *GAPDH* cDNA was also amplified as an endogenous control using 5′-GTC CAC TGG CGT CTT CAC CA-3′ and 5′-GTG GCA GTG ATG GCA TGG AC-3′ primers. The first step of the thermocycle was an initial denaturation and enzyme activation at 95 °C for 15 min. It was followed by 20 cycles of 94 °C for 30 s, 60 °C for 30 s and 72 °C for 1 min. After final extension (72 °C for 10 min), the samples were separated on 1.5% agarose gel and visualized by ethidium bromide staining.

### 4.8. qPCR Analysis

Quantitative qPCR assay was performed in 20 µL final volume containing 5 µL 20× diluted cDNA, 1× PowerUp^TM^ SYBR^TM^ Green Master Mix, 0.5 µM forward and reverse primers, using the QuantStudio 12K Flex Real-Time PCR System (Thermo Fisher Scientific, Waltham, MA, USA). *SCD5A*, *SCD5B* and *SCD1* sequences were amplified by 5′-ATG GAA ACC GGC CCT ATG AC-3′/5′-CCC CAG CCA GCA CAT GAA AT-3′, 5′-GTG AGA TGC TTC GTG AAT GGC-3′/5′-CCT CCA GGG ACA CAG AAA GAG-3′ and 5′-CTG GCC TAT GAC CGG AAG AAA-3′, 5′-GAC CCC AAA CTC ATT CCA TAG G-3′ primer pairs, respectively. *GAPDH*, *Actin* and *Tubulin* cDNAs were also amplified as endogenous controls using 5′-GTC CAC TGG CGT CTT CAC CA-3′/5′-GTG GCA GTG ATG GCA TGG AC-3′, 5′-CTG GTG CCT GGG GCG-3′/5′-AGC CTC GCC TTT GCC GA-3′ and 5′-AAG TTC GCA CTG GCA CCT AC-3′/5′-AAC CAA GAA GCC CTG GAG AC-3′ primer pairs, respectively. For increased reliability, an RT negative control of each sample was also analyzed in addition to DNase I digestion. The first step of the thermocycle was an initial denaturation and enzyme activation at 95 °C for 2 min. It was followed by 40 cycles of 95 °C for 15 s, 55 °C for 15 s and 72 °C for 1 min, measurement of the fluorescent signal was carried out during annealing. Reactions were performed in triplicates, a reaction mixture with RNase-free water instead of template cDNA was employed as a non-template control. Relative expression levels were calculated as 2^−Δ*C*T^, where Δ*C*_T_ values corresponded to the difference of the *C*_T_-values of the endogenous control and target genes.

### 4.9. Statistics

Immunoblots were evaluated by densitometry using the Image Studio^®^ 5.2 software (LI-COR Biotechnology, Lincoln, NE, USA), and are shown as relative band densities normalized to actin as a reference or on a percentage scale. Data are presented in the diagrams as the mean values ± S.D. and were compared by ANOVA with the Tukey’s multiple comparison post hoc test, using the GraphPad Prism 6.0 software (GraphPad Software, Boston, MA, USA). Differences with a *p* < 0.05 value were considered to be statistically significant.

## 5. Conclusions

Due to the limited expression of a few tissue types, it is possible that research on SCD5 has lagged significantly behind that of the more ubiquitous other human desaturase isoform. The existence of the two TVs of SCD5 has long been known, but remained largely disregarded in most studies. However, recent findings shed light on the importance of SCD5 expression and AS in tumor progression and metastasis, and their potential as prognostic factors and predictive biomarkers for tumor therapy. This is why we felt the need to compare the distribution of SCD5A and SCD5B in human tissues, and to elucidate the molecular background of the disproportionate AS of the primary transcript. This gap-filling research was also complemented by the investigation of certain SNVs potentially affecting the AS of *SCD5*. We have demonstrated the expression of both *SCD5A* and *SCD5B* forms and the general dominance of the former in several human tissues, provided evidence for the markedly different binding probabilities of the alternative splice acceptor sites underlying the phenomenon, and revealed the impact of various human gene variants on *SCD5* AS causing remarkable changes in the relative expression levels of the TVs, even in the dominance of SCD5B, in one case. We believe that our findings will facilitate further studies on tumor-related metabolic reprogramming of diagnostic, prognostic and therapeutic significance.

## Figures and Tables

**Table 1:** Location of SNVs affecting the AB donor site or the A or B acceptor site in the *SCD5* gene and their effect predicted by the VEP program. The exact sequence context is provided in Figure S3.

SNV ID	Wt	Mut	Affected Exons	Change in		Predicted Consequence in		Impact
	Allele			Amino Acid	Codon	Protein	mRNA	
**rs145164872**	A	G	3/4B	Asn/Asp	Aat/Gat	missense	srv	moderate
			3/4A	Lys/Glu	Aag/Gag			moderate
**rs1430176385**	G	A	3/4B	–	–	–	sav	high
**rs140750150**	C	T	3/4B	Thr/Ile	aCa/aTa	missense	srv	moderate
		G		Thr/Arg	aCa/aGa	missense	srv	moderate
**rs1250613148**	C	A	3/4A	–	–	–	spYv, srv	low
**rs1225904796**	C	T	3/4A	–	–	–	spYv, srv	low
**rs1011850309**	G	C	3/4A	Lys/Asn	aaG/aaC	missense	srv	moderate
		A		Lys	aaG/aaA	synonymous	srv	low

wt: wild type; mut: minor allele; srv: splice region variant; sav: splice acceptor variant; spYv: splice poly-Y tract variant.

## Data Availability

All data are available in the main text or the Supplementary Materials. Research materials used in the article can be requested from authors. This study includes no data deposited in external repositories.

## Supplementary Materials

The supporting information can be downloaded at: https://www.mdpi.com/article/10.3390/ijms24076517/s1.
