# Supplementary material for: Molecular Basis of Unequal Alternative Splicing of Human SCD5 and Its Alteration by Natural Genetic Variations
Source: Int J Mol Sci. 2023 Mar 30;24(7):6517. doi: 10.3390/ijms24076517 (PMC10095032; doi:10.3390/ijms24076517)
Supplement: Supplementary file 1 [file ijms-24-06517-s001.zip › Figures S1-S6_proof.pdf]

Molecular Basis of Unequal Alternative Splicing of Human SCD5 and Its Alteration by Natural Genetic Variations

Gabriella Orosz [1], Luca Szabó [1], Szanna Bereti [1], Veronika Zámbó [1], Miklós Csala [1,*] and Éva Kereszturi [1,*]

[1] Semmelweis University, Department of Molecular Biology, Budapest, Hungary; orosz.gabriella@med.semmelweis-univ.hu (G.O.); saboluca@gmail.com (L.S.); szanna.bereti@gmail.com (S.B.); zambo.veronika@med.semmelweis-univ.hu (V.Z.);

* Correspondence: csala.miklos@semmelweis.hu (M.C.) and kereszturi.eva@med.semmelweis-univ.hu (É.K.)

Supplementary Figures

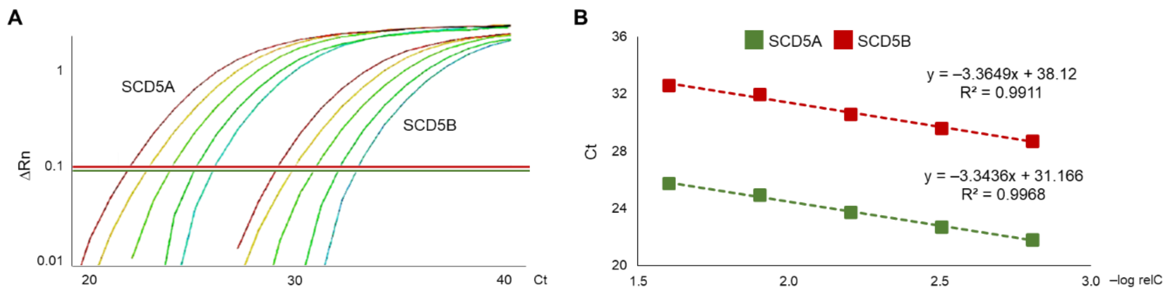

Figure S1. Optimization of quantitative measurement of *SCD5A* and *SCD5B* mRNA levels. **A**. The Ct value of the amplification curves made with *SCD5A* and *SCD5B* specific qPCR from the half dilution series of the human brain cDNA sample was plotted as a function of the relative fluorescence. Green line: threshold for *SCD5A*; red line: threshold for *SCD5B*. Representative results of three parallel experiments are shown. **B**. Comparison of *SCD5A* and *SCD5B* specific qPCR reaction efficiency. The negative logarithm of the relative concentrations was plotted as a function of the Ct values. Data are presented as mean values. Due to their low value, S.D.s are found in Table S1 instead of the diagram. (Ct: cycle threshold; y: equation of straight line; R^2: coefficient of determination; –log relC: negative logarithm of relative cDNA concentration)

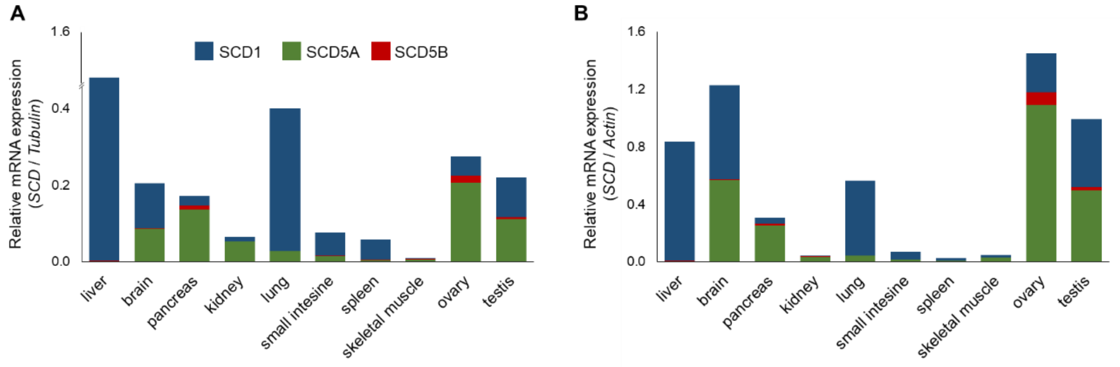

Figure S2. Cumulated relative gene expression of *SCD*s in human tissues normalized with *Tubulin* (**A**) and *Actin* (**B**) controls. Gene expression of *SCD5A*, *SCD5B* and *SCD1* was measured together with two additional endogenous controls, *Tubulin* and *Actin*, in ten different human tissues and then plotted cumulatively. Experiments were performed in triplicate. The S.D. values are presented in Tables S5.

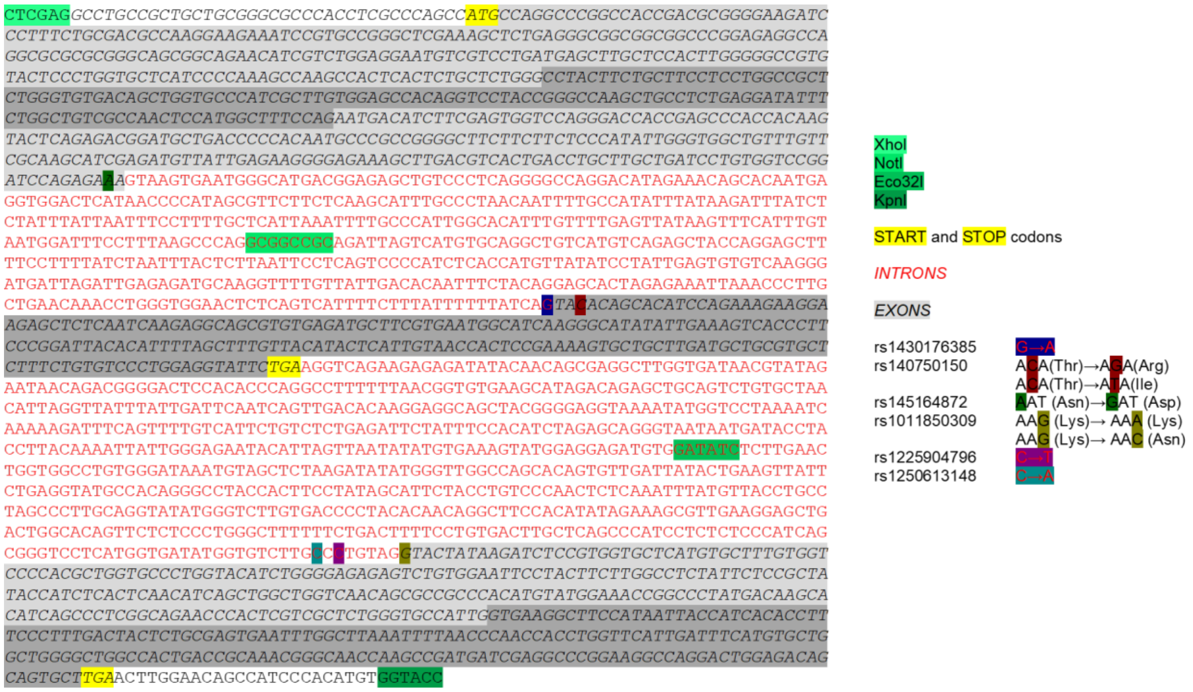

Figure S3. Sequence of the SCD5 minigene. The restriction endonuclease recognition sites used for cloning were highlighted in green, the stop and start codons in yellow. Exons have a gray background, intron sequences are written in red. Positions of SNVs affecting splice sites are highlighted in different colors in the sequence.

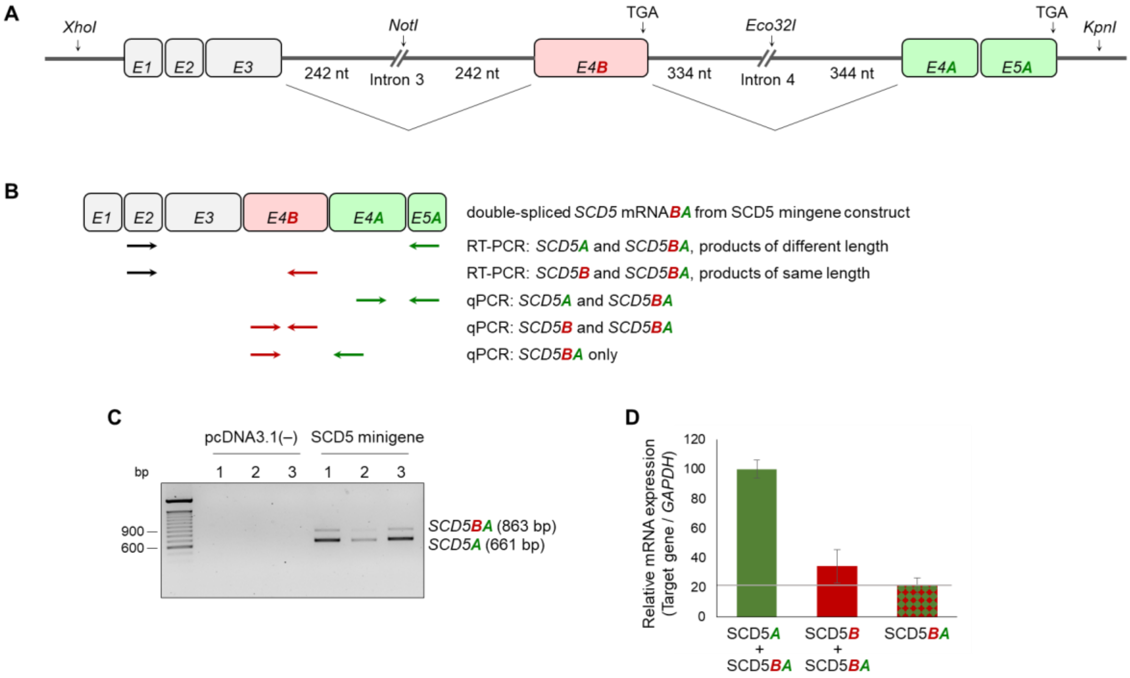

Figure S4. Unconventional *in vitro* splicing of *SCD5*. **A.** Schematic representation of unconventional minigene-driven *SCD5* splicing. Exons are numbered and marked with rectangles. *SCD5A* and *SCD5B* specific, and shared exons are shown in green, red, and gray, respectively. Introns are depicted with straight lines. The length of the segments cloned from 5’ and 3’ regions of introns 3 and 4 are also given. The stop codons and restriction endonuclease cleavage sites are indicated. **B.** Binding sites of the RT-PCR and qPCR primers on the *SCD5BA* artificial template. Black, green, and red arrows indicate primers specific for shared, A and B specific exons. **C.** The fragments amplified in *SCD5A* specific RT-PCR reaction were separated on 2% agarose gel. **D.** Relative mRNA expression of *SCD5A*, *B* and *BA* was measured by qPCR in HEK293T cells transfected with the SCD5 minigene as described in *Materials in Methods*. *GAPDH* served as an endogenous control.

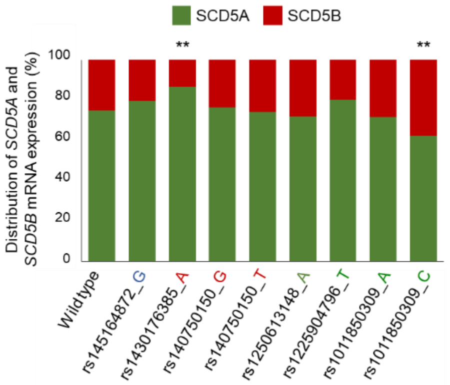

Figure S5. Effect of SNVs on unequal splicing of *SCD5* mRNA levels *in vitro*. HEK293T cells were harvested and processed 24 h after transfection with SCD5 minigene variants. The mRNA expression of *SCD5A* and *SCD5B* was measured by qPCR as described in *Materials and Methods*. *GAPDH* gene expression served as a control. The distribution of *SCD5* transcription is presented on a percentage scale. The S.D. values are shown in Table S7. Statistical analysis was performed with the Tukey-Kramer Multiple Comparisons Test. $**p < 0.01$.

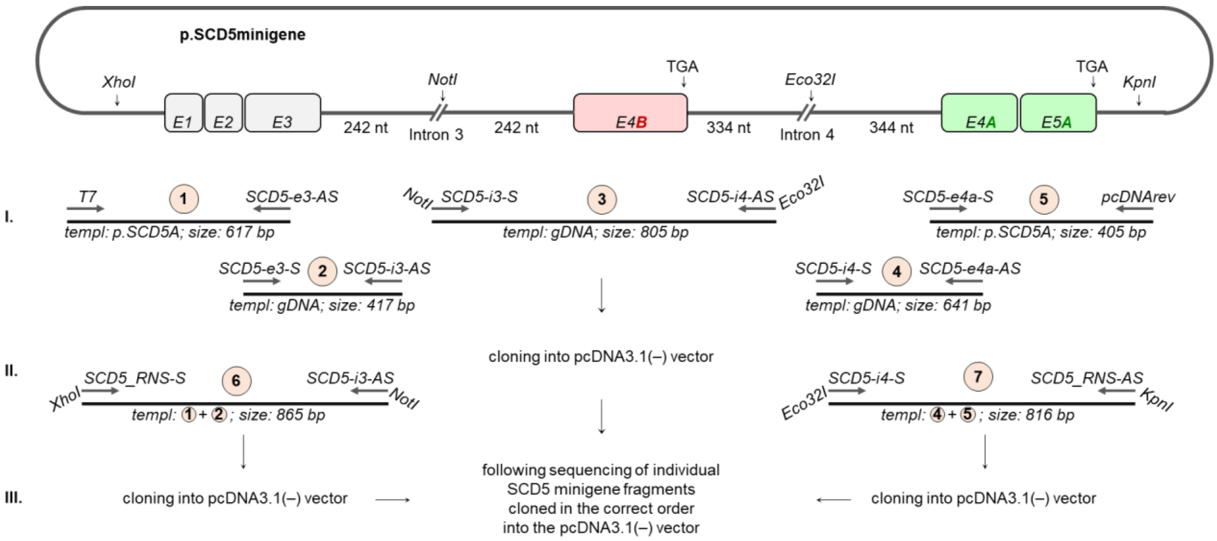

Figure S6. Cloning process of the SCD5 minigene. The order of amplification, the name and position of primer pairs, the template, and the size of the fragments are indicated. The primer sequences are shown in Table S8. The order of the three main steps of the cloning process is indicated by Roman numerals. Exons are numbered and marked with rectangles. *SCD5A* and *SCD5B* specific, and shared exons are shown in green, red, and gray, respectively. Introns are depicted with straight lines. The length of the segments cloned from 5’ and 3’ regions of introns 3 and 4 are also given. The stop codons, as well as restriction endonuclease cleavage sites are indicated. (bp: base pair; nt: nucleotide; templ.: template)
