# Supplementary material for: Molecular Basis of Unequal Alternative Splicing of Human SCD5 and Its Alteration by Natural Genetic Variations
Source: Int J Mol Sci. 2023 Mar 30;24(7):6517. doi: 10.3390/ijms24076517 (PMC10095032; doi:10.3390/ijms24076517)
Supplement: Supplementary file 1 [file ijms-24-06517-s001.zip › Tables S1-S9_rev.pdf]

Molecular Basis of Unequal Alternative Splicing of Human SCD5 and Its Alteration by Natural Genetic Variations

Gabriella Orosz [1], Luca Szabó [1], Szanna Bereti [1], Veronika Zámbó [1], Miklós Csala [1,*] and Éva Kereszturi [1,*]

[1] Semmelweis University, Department of Molecular Biology, Budapest, Hungary; orosz.gabriella@med.semmelweis-univ.hu (G.O.); saboluca@gmail.com (L.S.); szanna.bereti@gmail.com (S.B.); zambo.veronika@med.semmelweis-univ.hu (V.Z.)
* Correspondence: csala.miklos@semmelweis.hu (M.C.) and kereszturi.eva@med.semmelweis-univ.hu (É.K.)

Supplementary Tables

Table S1. Standard deviation values of Figure S1B.

Dilution (rel c)	–log(rel c)	Ct ± S.D.	
		SCD5A	SCD5B
**640**	2.8062	21.8307 ± 0.0431	28.7317 ± 0.1735
**320**	2.5051	22.7080 ± 0.0130	29.6060 ± 0.1310
**160**	2.2041	23.7545 ± 0.0403	30.5980 ± 0.1861
**80**	1.9031	24.9387 ± 0.0530	31.9597 ± 0.0798
**40**	1.6021	25.7480 ± 0.0638	32.6195 ± 0.1407

Table S2. Standard deviation values of Figure 2D.

Tissue	Relative expression values ± S.D.		
	SCD5A	SCD5B	SCD1
**liver**	0.0005 ± 3.94E-05	0.0001 ± 4.56E-05	0.1680 ± 4.73E-03
**brain**	0.1294 ± 4.42E-03	0.0017 ± 1.26E-04	0.2031 ± 1.53E-02
**pancreas**	0.0706 ± 4.62E-03	0.0052 ± 1.82E-03	0.0134 ± 1.69E-03
**kidney**	0.0136 ± 9.33E-04	0.0004 ± 1.16E-04	0.0028 ± 1.29E-04
**lung**	0.0113 ± 1.62E-03	0.0004 ± 2.28E-05	0.1609 ± 5.08E-03
**small intesine**	0.0034 ± 1.71E-04	0.0002 ± 6.53E-05	0.0163 ± 3.41E-03
**spleen**	0.0040 ± 1.68E-04	0.0002 ± 5.61E-05	0.0190 ± 3.76E-04
**skeletal muscle**	0.0003 ± 5.39E-05	0.0000 ± 8.15E-06	0.0002 ± 1.66E-05
**ovary**	0.1890 ± 1.67E-02	0.0290 ± 1.72E-03	0.0668 ± 9.78E-03
**testis**	0.0319 ± 5.04E-03	0.0047 ± 1.88E-04	0.0710 ± 1.66E-02

Table S3. Standard deviation values of Figure 2E.

Tissue	% of total SCD5 ± S.D.	
	SCD5A	SCD5B
liver	75.19 ± 10.06	24.81 ± 10.06
brain	98.73 ± 0.11	1.27 ± 0.11
pancreas	93.65 ± 0.85	6.35 ± 0.85
kidney	97.54 ± 0.38	2.46 ± 0.38
lung	96.60 ± 0.62	3.40 ± 0.62
small intesine	92.42 ± 1.32	7.58 ± 1.32
spleen	90.65 ± 7.65	9.35 ± 7.65
skeletal muscle	88.91 ± 4.01	11.09 ± 4.01
ovary	90.40 ± 3.23	9.60 ± 3.23
testis	93.04 ± 5.21	6.96 ± 5.21

Table S4. Standard deviation values of Figure 2F.

Tissue mRNA	% of total SCD ± S.D.		
	SCD5A	SCD5B	SCD1
**liver**	0.38 ± 0.13	0.13 ± 0.08	99.49 ± 0.14
**brain**	42.69 ± 0.55	0.55 ± 0.04	56.76 ± 3.08
**pancreas**	80.56 ± 5.45	5.45 ± 0.70	13.99 ± 0.55
**kidney**	82.28 ± 2.07	2.07 ± 0.27	15.65 ± 2.21
**lung**	6.67 ± 0.24	0.24 ± 0.07	93.09 ± 0.85
**small intesine**	18.06 ± 1.50	1.50 ± 0.42	80.44 ± 2.66
**spleen**	14.73 ± 1.18	1.18 ± 0.41	84.09 ± 6.45
**skeletal muscle**	60.52 ± 7.69	7.69 ± 3.39	31.78 ± 5.16
**ovary**	72.21 ± 7.61	7.61 ± 2.29	20.18 ± 2.50
**testis**	46.23 ± 3.23	3.23 ± 1.97	50.54 ± 6.12

Table S5. Standard deviation values of Figure S2.

Tissue	Relative expression values ± S.D.					
	SCD5A		SCD5B		SCD1	
	Actin	Tubulin	Actin	Tubulin	Actin	Tubulin
liver	0.0039 ± 1.45E-03	0.0047 ± 5.20E-04	0.0015 ± 5.06E-04	0.0026 ± 3.93E-04	0.8315 ± 1.75E-01	1.2756 ± 2.18E-01
brain	0.5686 ± 1.48E-01	0.0859 ± 4.34E-03	0.0065 ± 1.01E-03	0.0012 ± 2.03E-04	0.6552 ± 6.60E-02	0.1180 ± 1.80E-02
pancreas	0.2513 ± 3.27E-02	0.1370 ± 2.73E-02	0.0143 ± 1.81E-03	0.0102 ± 1.30E-03	0.0413 ± 3.37E-03	0.0250 ± 3.74E-03
kidney	0.0357 ± 6.81E-03	0.0522 ± 2.64E-03	0.0007 ± 7.24E-05	0.0014 ± 1.31E-04	0.0055 ± 7.27E-05	0.0104 ± 7.63E-04
lung	0.0419 ± 5.70E-03	0.0271 ± 2.67E-03	0.0017 ± 3.42E-04	0.0008 ± 4.29E-04	0.5225 ± 8.17E-02	0.3729 ± 2.75E-02
small intesine	0.0142 ± 6.91E-04	0.0144 ± 4.22E-03	0.0012 ± 4.16E-04	0.0014 ± 6.05E-04	0.0560 ± 3.92E-03	0.0609 ± 1.54E-02
spleen	0.0052 ± 5.41E-04	0.0043 ± 2.56E-04	0.0003 ± 1.48E-04	0.0010 ± 1.20E-04	0.0196 ± 2.19E-03	0.0530 ± 1.01E-02
skeletal muscle	0.0276 ± 4.76E-03	0.0061 ± 6.40E-04	0.0026 ± 9.42E-04	0.0012 ± 2.15E-04	0.0162 ± 8.42E-03	0.0028 ± 1.31E-03
ovary	1.0894 ± 3.32E-01	0.2071 ± 3.53E-02	0.0903 ± 2.34E-02	0.0172 ± 1.27E-03	0.2728 ± 8.78E-02	0.0516 ± 6.81E-03
testis	0.4978 ± 1.25E-01	0.1119 ± 9.70E-03	0.0206 ± 5.57E-03	0.0046 ± 4.90E-04	0.4726 ± 1.75E-01	0.1040 ± 1.67E-02

Table S6. Standard deviation values of Figure 4C and E.

SCD5 expression		% of total SCD5 ± S.D.	
		SCD5A	SCD5B
mRNA	endogenous	69.83 ± 4.06	30.17 ± 4.06
	minigene	69.70 ± 0.74	30.30 ± 0.74
protein	minigene	75.38 ± 10.27	24.62 ± 10.27

Table S7. Standard deviation values of Figure S5 and Figure 6B.

	% of total SCD5 mRNA ± S.D.		% of total SCD5 protein ± S.D.	
	**SCD5A**	**SCD5B**	**SCD5A**	**SCD5B**
**wild type**	74.87 ± 1.50	25.13 ± 1.50	83.31 ± 6.57	16.69 ± 6.57
**rs145164872_G**	79.76 ± 1.31	20.24 ± 1.31	83.03 ± 6.83	16.97 ± 6.83
**rs1430176385_A**	86.65 ± 0.88	13.35 ± 0.88	99.16 ± 0.57	0.84 ± 0.57
**rs140750150_G**	76.64 ± 5.08	23.36 ± 5.08	90.96 ± 4.14	9.04 ± 4.14
**rs140750150_T**	74.39 ± 1.14	25.61 ± 1.14	87.57 ± 4.20	12.43 ± 4.20
**rs1250613148_A**	72.02 ± 0.49	27.98 ± 0.49	83.11 ± 2.79	16.89 ± 2.79
**rs1225904796_T**	80.41 ± 3.01	19.59 ± 3.01	87.02 ± 3.84	12.98 ± 3.84
**rs1011850309_A**	71.74 ± 1.09	28.26 ± 1.09	68.24 ± 6.47	31.76 ± 6.47
**rs1011850309_C**	62.53 ± 5.53	37.47 ± 5.53	24.56 ± 4.17	75.44 ± 4.17

Table S8. Sequence and annealing temperature of cloning primers. Restriction endonuclease recognition sites are highlighted in underlined bold letters.

**Primer name**	**Sequence 5' → 3'**	**T_m (°C)**
**T7**	TAATACGACTCACTATAGGG	48
**SCD5_e3-AS**	TTCTCCCCTTCTCAATAACA	48
**SCD5_e3-S**	GAGCCCACCACAAGTACTCA	54
**SCD5_i3-AS**	ATGACACTGGGCTTAAAGGAA	50
**SCD5_i3-S-*Not*I**	AAATTT**GCGGCCGC**AGATTAGTCATGTGCAGGCTGT	53
**SCD5_i4-AS-*Eco*32I**	AAATTT**GATATC**CACATCTCCTCCATACTTTCACA	53
**SCD5_i4-S**	TTACCAAGGTGAGTTAGTACAGAA	52
**SCD5_e4a-AS**	GTTTCCATACATGTGGGCGG	54
**SCD5_e4a-S**	CTTGGCCTCTATTCTCCGC	53
**pcDNArev**	TAGAAGGCACAGTCGAGG	50
**SCD5_RNS-S-*Xho*I**	AAATTT**CTCGAG**GCCTGCCGCTGCTG	49
**SCD5_i3-AS-*Not*I**	AAATTT**GCGGCCGC**CTGGGCTTAAAGGAAATCCA	50
**SCD5_i4-S-*Eco*32I**	AAATTT**GATATC**TCTTGAACTGGTGGCCTGTG	54
**SCD5_RNS-AS-*Kpn*I**	AAATTT**GGTACC**ACATGTGGGATGGCTGTTC	51

Table S9. Sequence and annealing temperature of mutagenic primers. Nucleotide exchanges are highlighted in underlined bold lowercase letters.

Mutagenic primer name	Sequence 5' → 3'	T_m (°C)
**rs1430176385G>A-S**	TTTTTTATCA<u>**a**</u>TACACAGCAC	57
**rs1430176385G>A-AS**	TAAAGAAAATGACTGAGAGTTC	57
**rs140750150C>G-S**	TTTATCAGTA<u>**g**</u>ACAGCACATC	56
**rs140750150C>T-S**	TTTATCAGTA<u>**t**</u>ACAGCACATCC	56
**rs140750150C>G/T-AS**	AAATAAAGAAAATGACTGAGAG	56
**rs145164872A>G-S**	GATCCAGAGA<u>**g**</u>AGTAAGTGAATGGG	63
**rs145164872A>G-AS**	CGGACCACAGGATCAGCA	63
**rs1011850309G>A-S**	TGCCCTGTAG<u>**a**</u>TACTATAAGATC	59
**rs1011850309G>C-S**	TGCCCTGTAG<u>**c**</u>TACTATAAGATC	57
**rs1011850309G>A/C-AS**	AGACACCATATCACCATG	59
**rs1225904796C>T-S**	GTGTCTTGCC<u>**t**</u>TGTAGGTACTATAAG	60
**rs1225904796C>T-AS**	CATATCACCATGAGGACC	60
**rs1250613148C>A-S**	TGGTGTCTTG<u>**a**</u>CCTGTAGGTA	65
**rs1250613148C>A-AS**	TATCACCATGAGGACCCG	65
